# Regime Shifts and Weakened Environmental Gradients in Open Oak and Pine Ecosystems

**DOI:** 10.1371/journal.pone.0041337

**Published:** 2012-07-24

**Authors:** Brice B. Hanberry, Dan C. Dey, Hong S. He

**Affiliations:** 1 Department of Forestry, University of Missouri, Columbia, Missouri, United States of America; 2 USDA Forest Service, Northern Research Station, University of Missouri, Columbia, Missouri, United States of America; University of Western Australia, Australia

## Abstract

Fire suppression allows tree species that are intolerant of fire stress to increase their distribution, potentially resulting in disruption of historical species-environmental relationships. To measure changes between historical General Land Office surveys (1815 to 1850) and current USDA Forest Inventory and Assessment surveys (2004 to 2008), we compared composition, distribution, and site factors of 21 tree species or species groups in the Missouri Ozarks. We used 24 environmental variables and random forests as a classification method to model distributions. Eastern redcedar, elms, maples, and other fire-sensitive species have increased in dominance in oak forests, with concurrent reductions by oak species; specific changes varied by ecological subsection. Ordinations displayed loss of separation between formerly distinctive oak and fire-sensitive tree species groups. Distribution maps showed decreased presence of disturbance-dependent oak and pine species and increased presence of fire-sensitive species that generally expanded from subsections protected from fire along rivers to upland areas, except for eastern redcedar, which expanded into these subsections. Large scale differences in spatial gradients between past and present communities paralleled reduced influence of local topographic gradients in the varied relief of the Missouri Ozarks, as fire-sensitive species have moved to higher, drier, and sunnier sites away from riverine corridors. Due to changes in land use, landscapes in the Missouri Ozarks, eastern United States, and world-wide are changing from open oak and pine-dominated ecosystems to novel oak-mixed species forests, although at fine scales, forests are becoming more diverse in tree species today. Fire suppression weakened the influence by environmental gradients over species dominance, allowing succession from disturbance-dependent oaks to an alternative state of fire-sensitive species. Current and future research and conservation that rely on historical relationships and ecological principles based on disturbance across the landscape will need to incorporate modern interactions among species for resources into management plans and projections.

## Introduction

Widespread clearing of interior eastern United States forests during the turn of the 20th century, followed by gradual reforestation and fire suppression, have produced forests with different composition and structure than pre-settlement oak and pine savannas, woodlands, and forests [1]–[3]. A heavy canopy of oaks developed from the regrowth of forests following the period of intensive harvesting. Fully-stocked forests and subsequent fire suppression allowed a relatively dense understory of fire-sensitive and increasingly shade-tolerant species to develop [3]. Consequently, dense forests with multi-layered canopies became more widespread in areas that were not converted to pasture or cropfields while open oak and pine savannas and woodlands were relegated to remnant portions of the landscape.

Ecological relationships among species distributions, dominance, and environmental factors may not be as pronounced today as in historical times [4]–[5]. Depending on the disturbance regime, silvical characteristics allowed species to dominate on xeric, mesic, or hydric sites in historical forests. Because of fire suppression and abandonment of agricultural fields, species traditionally associated with floodplains increased in upland forests [6], whereas eastern redcedar (*Juniperus virginiana*) encroached into glades, savannas, and old fields [7]–[8]. Throughout eastern North America, many sub-dominant species such as red maple (*Acer rubrum*) were released from their previously realized distributions to create essentially novel communities dominated by fire-intolerant species [9]–[11]. Increased distributions by species that are not competitive during a fire regime may disrupt historical species-environmental relationships, due to a lack of fire interaction with existing site conditions, such as soil moisture and fertility [12]–[15].

**Figure 1:**
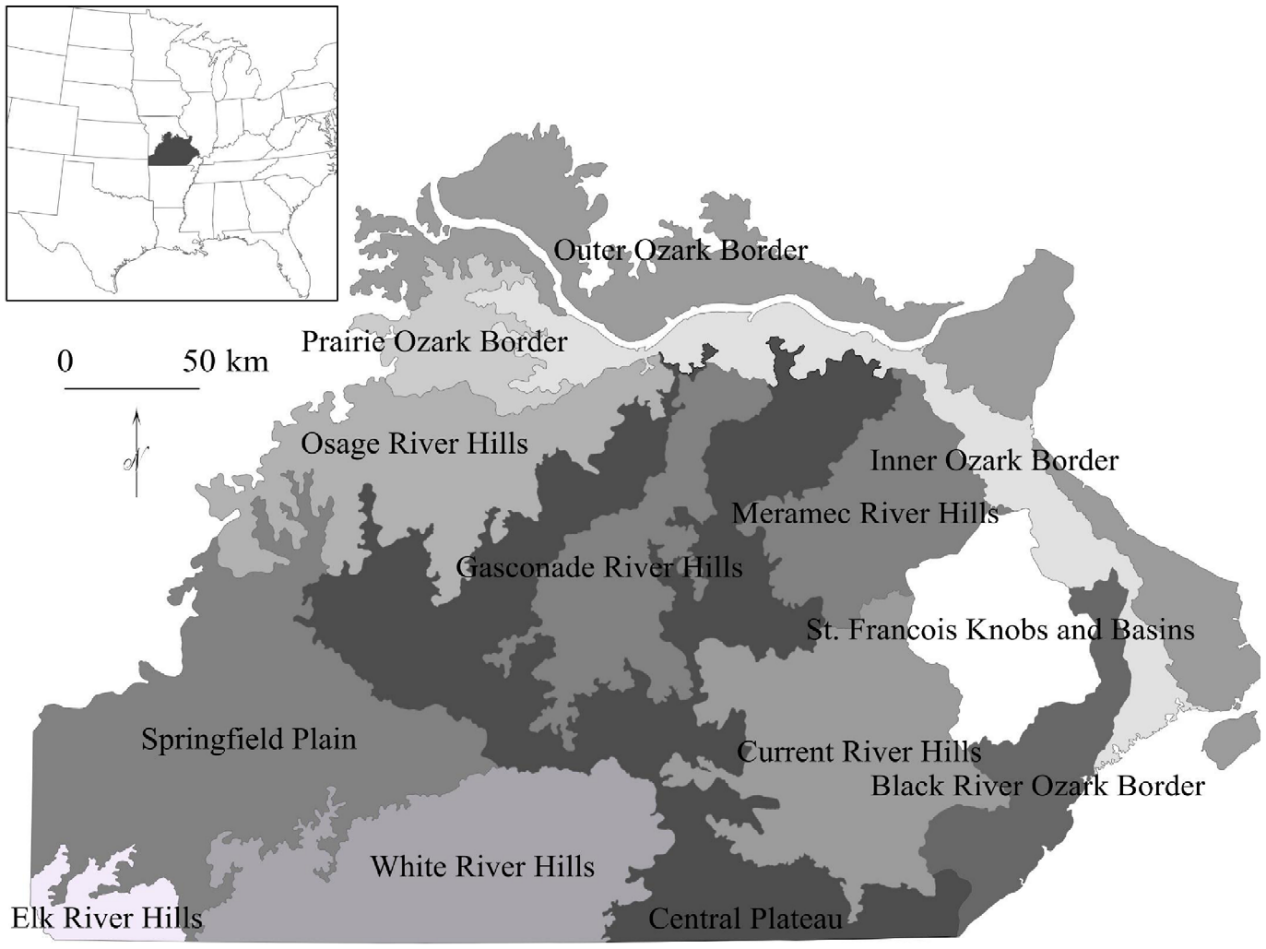
Ecological subsections of the study extent, the Missouri Ozarks ecological section.

Climate, fire, topography, soils, and geology interacted with human land use to produce historical landscapes, including the Missouri Ozark Highlands, a mosaic of prairies, oak and pine savannas and woodlands, and mesic mixed hardwood forests [1], [16]. Although climate during the Little Ice Age, which ended in mid to late 1800 s, was cooler than today, fires were more common [17]. The historical fire regime largely reflected changes in human populations and cultures. Fires were more frequent on topographically flat areas such as the Central Plateau and Springfield Plain ecological subsections (i.e., spatial divisions based on ecological similarities; [18]; Figure 1) in the western and central Ozarks [19]. In areas characterized by deeply dissected topography such as the Current River Hills subsection, fire was limited by physical land features that acted as barriers to its spread, and hence its frequency at any given site. Fire-intolerant species or less fire-tolerant white oak (*Quercus alba*) were able to dominate protected northeastern aspects, lower slope positions, and riparian areas, for example, the fire shadow in the rugged hills of the Current River watershed [20].

A common change in fire regime occurred throughout eastern North America. Fires were a dominant factor determining the distribution of species, their abundance, competitive relations, and vegetative structure during 10,000 years or more [21] that Native Americans inhabited eastern forests of the United States [17], [22]. European settlers initially maintained or in many cases increased the frequency of fire compared to Native American occupation until regional development when fires become less prevalent or even rare due to suppression, changes in fuels, and increases in anthropogenically-created barriers to fire spread [17,20,22–23). With increased occupation and development and the dawning of the conservation era in the 1930 s came increased efforts and technology to prevent and suppress wildfires. For 50 years or more wildfires have been practically eliminated from the Ozark Highlands and currently, about 2,500 wildfires are reported in Missouri in a typical year; most are less than four hectares in size and they burn less than 20,000 hectares statewide [24].

European settlement resulted in loss of the historical fire regime after fire suppression and extensive forest clearing for agriculture and timber harvesting. These changes in land use modified successional pathways and species distributions, abundance, and dominance. By comparing historical and current forests, we expected to identify mesophication (i.e., stable forests of fire-sensitive and increasingly shade-tolerant species replacing disturbance-dependent oak and pine ecosystems; [3]) of forests and concomitant weakening of species-environmental relationships without fire to organize species along environmental gradients. To measure changes between historical (General Land Office surveys; 1815 to 1850) and current (USDA Forest Inventory and Assessment surveys; 2004 to 2008) forests, we compared composition, distribution, and species-environmental relationships of 21 tree species or groups across the Ozark Highlands of Missouri. Specifically, we intend to 1) identify species composition (relative abundance) changes in the Ozarks and within smaller subsections, 2) depict shifts in species spatial location over time, 3) delineate communities along environmental gradients and determine their heterogeneity, and 4) quantify historical and current associations between tree species and site factors to address if species-environmental relationships have been de-coupled, at least at a small scale, to provide information about the future direction of oak and pine ecosystems in the Ozark Highlands and elsewhere without fire disturbance.

## Methods

### Tree Surveys and Composition

The General Land Office (GLO) developed the Public Land Survey System of townships and ranges in 1812 [25]. Public lands were divided into townships measuring 9.6 km on a side, and townships were divided into 1.6×1.6 km sections. Surveyors recorded species, distance, bearing, and diameter for two to four bearing trees at survey points at the corners and middle of each section line (i.e., every 0.8 km) and recorded trees encountered along the section lines. We selected about 285,000 trees, surveyed primarily between 1815 to 1850, in the Ozark Highlands section of Missouri ([18], Figure 1) from the GLO dataset (J. Harlan, Geographic Resources Center, http://msdis.missouri.edu).

The USDA Forest Service Forest Inventory and Analysis (FIA) program collects numerous tree measurements for all trees at plots that are visited on a five year cycle. We used the latest complete cycle from 2004–2008. Available FIA plot locations are perturbed (i.e., location moved) to protect landowners. Therefore, for species distribution modeling, the USDA Forest Service matched environmental variables to plots without revealing plot location. There were about 3,800 plots (with 72,000 trees) that intersected our spatial units of soil polygons (9,073,300 ha total), thus there was about one plot every 2,400 ha in the Ozarks.

It is complicated to compare composition of GLO and FIA tree species, because FIA surveys are more complete inventories of the tree population including understory and overstory trees, whereas GLO surveyors used primarily overstory trees to monument survey points. Thus, we chose to remove less common species from comparison over the entire Ozarks (but not by ecological subsection in case of the appearance of novel species). Many of these were understory trees, such as flowering dogwood (*Cornus florida*), eastern redbud (*Cercis canadensis*), and common persimmon (*Diospyros virginiana*), representing about 0.5% of GLO trees and 6.5% of FIA trees. In GLO surveys, surveyors choosing species with greater longevity, species that were easy to blaze, those with thin bark and few lower branches [26], and species that were unusual or out of the ordinary. The line trees in GLO surveys were intended to be a record of trees encountered by the surveyor along the section lines, compared to bearing trees, which were selected by the surveyor. Therefore, differences between line tree percentage and bearing tree percentage may represent a range of normal variation due to bias in surveys.

We determined percent composition of 21 species groups, based on existing GLO categories (Table 1), as a rough measure of relative abundance. For GLO surveys, we used 60,000 line trees, which surveyors encountered along the section lines, and therefore were subject to less surveyor bias, and 225,000 bearing trees that were selected, recorded by bearing, and blazed. We also calculated line species composition by ecological subsection ([18], Figure 1). For FIA surveys, we used available plots (FIA DataMart, http://www.fia.fs.fed.us/tools-data) to determine species frequencies for the Ozarks and by subsection. We selected about 50,000 live trees that were ≥7.6 cm in diameter, which is the smallest diameter for trees in GLO surveys.

**Table 1:** Tree species/group composition for GLO (1815–1850) and FIA (2004–2008, ≥7.6 cm DBH) surveys in the Missouri Ozarks.

Species/group		GLO line		GLO bearing		FIA	
		Count	%	Count	%	Count	%
ashes	*Fraxinus americana, F. pennsylvanica*	407	0.68	2224	0.99	967	1.91
blackgum	*Nyssa sylvatica*	372	0.62	1624	0.72	652	1.29
black oaks	*Quercus velutina, also Q. falcata, Q. coccinea, Q. rubra*	14034	23.56	48326	21.47	10180	20.15
blackjack oak	*Quercus marilandica*	3450	5.79	18383	8.17	860	1.70
bottomland	*Populus* spp., *Salix* spp.	112	0.19	639	0.28	118	0.23
(cottonwood, willow)							
bur oak	*Quercus macrocarpa*	366	0.61	1160	0.52	34	0.07
cherries	*Prunus* spp.	34	0.06	206	0.09	487	0.96
chinkapin oak	*Quercus muehlenbergii*	70	0.12	624	0.28	993	1.97
eastern redcedar	*Juniperus virginiana*	50	0.08	314	0.14	4472	8.85
elms	*Ulmus alata, U. americana, U. rubra*	878	1.47	4844	2.15	2048	4.05
hackberry	*Celtis occidentalis*	426	0.72	1558	0.69	414	0.82
hickories	*Carya cordiformis, C. glabra, C. laciniosa, C. ovata, C. texana, C. tomentosa*	3784	6.35	11962	5.31	5730	11.34
maples	*Acer rubrum, A. negundo, A. saccharum, A. saccharinum*	402	0.67	2420	1.08	1510	2.99
mesic	*Tilia americana, Carpinus caroliniana, Robinia pseudoacacia, Betula* spp., *Gleditsia triacanthos, Morus alba* and *M. rubra, Liquidambar styraciflua*	370	0.62	1866	0.83	782	1.55
(American basswood, American hornbeam, black locust, birch, honeylocust, red and white mulberry, sweetgum)							
pin oak	*Quercus palustris*	316	0.53	1496	0.66	35	0.07
post oak	*Quercus stellata*	12154	20.40	53479	23.76	6745	13.35
sassafras	*Sassafras albidum*	13	0.02	117	0.05	386	0.76
shortleaf pine	*Pinus echinata*	5591	9.38	13925	6.19	3276	6.48
sycamore	*Platanus occidentalis*	328	0.55	1958	0.87	294	0.58
walnuts	*Juglans nigra, J. cinerea*	478	0.80	2308	1.03	959	1.90
white oak	*Quercus alba*	15943	26.76	55649	24.72	9588	18.97

The GLO line trees were encountered by the surveyor and the GLO bearing trees were selected by surveyors.

### Species Distribution Modeling

We used Soil Survey Geographic (SSURGO) Database (Natural Resources Conservation Service, http://soildatamart.nrcs.usda.gov) polygons as our spatial unit, and split polygons that were backslopes (i.e., slopes greater than 15%) based on exposed southwestern and protected northeastern aspects. After processing, there were about 835,000 polygons for the Missouri Ozarks (polygon mean area of 11 ha). Because discontiguous soil polygons share characteristics, our prediction unit was a unique zone (mean area of 131 ha) based on soil map unit (i.e., polygons with similar soil characteristics in a county), ecological land type [18], geology, and landform type (protected backslope, exposed backslope, and other).

We prepared 15 environmental variables from the SSURGO tables by soil map unit for each county. Variables included 1) landform type (bottomlands, protected backslope, exposed backslope, and uplands; determined by the Missouri Department of Conservation), 2) parent material kind (e.g., alluvium, colluvium, residuum), 3) origin (i.e., no limestone, limestone in combination, limestone), 4) drainage class (very poorly drained to excessively drained), 5) taxonomic order, 6) flooding frequency, and 7) restriction type (i.e., none, fragipan or claypan, bedrock). We also used 8) depth (cm) to either the bottom of the soil profile or soil restriction, after removing soil horizon layers below restrictions based on restrictive layer presence (corestriction table) and restrictive layers with suffixes (i.e., d, m, r, x). We then calculated 9) mean water holding capacity (cm/cm), 10) pH, 11) base saturation, 12) fragments (%), 13) organic matter (%), 14) clay (%), and 15) sand (%) to the depth and weighted values by component percentage.

From a 30 m DEM (digital elevation model), we calculated seven variables: elevation (m), slope (%), transformed aspect (1+ sin(aspect/180/3.14+0.79); [27]), solar radiation (0700 to1900 in 4 hour intervals on summer solstice for re-sampled 60 m DEM), topographic roughness [28], wetness convergence (ln(flow accum +1)/(tan(((slope deg)3.141593)/180));T. Dilts, http://arcscripts.esri.com), and topographic position index. We then calculated the mean value for each variable by prediction zones (based on map unit, ecological land type, geology, and landform type). We also joined ecological subsection and geology designations to each individual polygon.

We used GLO bearing trees for modeling, and removed about 4,000 trees in one area because they did not have a distance and bearing. There were about 213,000 GLO bearing trees and 67,000 FIA trees of these study species or species groups that intersected with the soil polygons. We joined the points or plots with each species or species group to generate the modeling sample, which were polygons that had survey points or plots. We then oversampled the minority class to reduce the inequality between the number of polygons with and without the tree species for modeling. We randomly selected 0.67 of polygons with the species, up to 2,500 polygons, for modeling, and held back the rest for prediction and validation. For pseudoabsences, we randomly selected up to 2,500 polygons without a recorded species presence from the modeling polygons (i.e., polygons with surveyed trees).

We applied two ensemble classification tree techniques: random forests and generalized (or gradient) boosting methods. For all species or species groups except shortleaf pine (*Pinus echinata*), we used random forests [29]–[30], a classification method based on bootstrap aggregation (bagging) by the majority vote of many trees grown using random samples of both predictor variables and training data. For shortleaf pine, we used generalized boosting classification [31]–[33], which is similar to random forests classification, but differs in that it is sequential and assigns greater weights (boosting) to misclassified cases, in order to create a better fitting model with the addition of each tree. Generalized boosting confined pine to a bounded distribution better than random forests, but otherwise showed less small-scale variation (Hanberry et al. in press).

We used the randomForest package [34] in R statistical software (R Development Core Team 2010), with the sampsize option (which is sampled without replacement*),* where we set the bag fraction, or subsampling rate, at 0.67 of the selected polygons with the species. We then specified 0.25 of that value for the selected polygons with unknown presence or absence of the tree species. We set the number of trees at 1,000 and the number of variables randomly sampled at each split as the square root of the number of predictors.

For generalized boosting methods, we used code by J. Leathwick and J. Elith [33] based on the R gbm package (G. Ridgeway, http://www.i-pensieri.com/gregr/gbm.shtml), with the Bernoulli distribution. We set the weight at 4, the bag fraction at 0.67, the shrinkage or learning rate at 0.005, and the interaction depth or tree complexity at 8. We allowed the program to determine the optimal number of trees based on AUC (not on true positive rate), a total of 2,650 trees for pine.

We used the ROCR package [35] in R to calculate the true positive rate over Receiver Operating Characteristic (ROC) curves for predictions. We determined the mean of predicted probabilities and compared predicted probabilities using Pearson’s correlation coefficient (SAS software, version 9.1, Cary, North Carolina; Proc Corr). We also grouped the predictions into 4 bins (0–25%, 25–50%, 50–75%, 75–100%) and mapped the distributions (please contact the authors for maps or GIS layers).

We examined environmental variable importance (i.e., permutation importance measure), ranked by the statistical method, to see if there were divergences between site variables for GLO and FIA surveys. We re-scaled variable importance values, by assigning the top value as 1, and dividing other values by the original value of the top variable. Although there was a drop-off in variable importance, it was not consistent and therefore, we focused on the top five variables from GLO and FIA modeling. We compared the mean values for recorded species.

### Ordinations

We explored communities through predicted probabilities from species distribution models, which represent response of species to environmental variables. We ran nonmetric multidimensional scaling (NMS) ordinations for each subsection, based on mean predicted probabilities by land type association (columns) and by species and survey type (rows), to visualize communities by subsection for eight subsections that had at least nine (to 35) ecological land type associations (PC-ORD version 4, MjM Software Design, Gleneden Beach, Oregon, USA). Sorensen/Bray-Curtis distance between species in the ordination scatter plot represents degree of similarity. We removed shortleaf pine, blackjack oak (*Quercus marilandica*), and bottomland species, if they had extremely low or variable predicted probabilities, creating considerable space around these species. For the Inner and Outer Border subsections, we removed shortleaf pine, blackjack oak, post oak (*Q*. *stellata*), chinkapin oak (*Q*. *muehlenbergii*), and blackgum (*Nyssa sylvatica*).

## Results

### Species Composition

Both records of trees along survey lines and trees from survey points suggested similar historic composition (Table 1). Mean absolute difference between percentage of trees surveyed for line and bearing trees in the GLO data was 0.8% and at most, 3.2% for shortleaf pine. For uncommon species, the greatest difference between line and bearing trees, as measured by the ratio of bearing tree percentage to line tree percentage, was 2.4 for chinkapin oak and sassafras (*Sassafras albidum*). Differences less than these we considered potentially inconsequential. In addition, bias in GLO surveys toward moderately-sized trees probably resulted in more records of overstory trees than species tolerant of the understory.

Composition of historical forests showed dominance by fewer species than in modern times (Table 1). For historical forests, white oak, black oak group (primarily *Quercus velutina,* but also *Q. falcata, Q. coccinea, Q. rubra*), and post oak were 71% of surveyed trees and currently they are 53% of surveyed trees. Shortleaf pine, hickories, and blackjack oak accounted for about 20% of both historical and current surveyed trees, due to doubled frequency of hickories that offset shortleaf pine and blackjack oak declines. Historically, elms were 1.5% of encountered trees and every other species was less than 1%. Within the group of associate species in the modern inventory, elms, maples, chinkapin oak, ashes, and walnuts comprised 13% of survey trees. Eastern redcedar increased from an insignificant presence to 9% in the FIA surveys.

Post oak and white oak had the largest relative decreases (7 to 8% each) based on a comparison of the percentage of trees from GLO line and FIA tree surveys. Shortleaf pine, blackjack oak, and the black oak group species experienced relatively moderate declines (3 to 4% each). Species that showed the largest increase since the GLO surveys were eastern redcedar (9%) and the hickories (5%), whereas elms, maples, chinkapin oak, ashes, and the mesic species group showed modest increases (increased by a factor of 2.4 to 17). The rest of the minor species generally increased relative to their proportion (e.g., uncommon sassafras and cherries increased by a factor of 17 to 35). Thus, the diversity of associated tree species increased since the GLO survey.

There were spatial shifts by subsection as well, for species ≥10% composition of GLO line trees or FIA trees by subsection (Table 2, five subsections not presented due to low tree counts). Generally, dominant post oak, black oak, white oak decreased, in some subsections by 50% or more, and hickories increased. In the Springfield Plain, blackjack oak decreased from 20% composition in the GLO line trees to 2% in the FIA surveys. In the Central Plateau, blackjack oak decreased from 13% to 5% composition. In the White River Hills, eastern redcedar increased from 1% to 19% composition. In the Gasconade River Hills, shortleaf pine increased from 3% to 11%, presumably due to planting. In the Current River Hills, shortleaf pine decreased from 54% to 15%. In the Knobs and Basins, shortleaf pine decreased from 12% to 7%. In the Black River Border, post oak increased from about 5% to 14%. In the Outer Border, maples increased from 2% to 10% and eastern redcedar increased from 0.06% to 11%. In the Inner Border, eastern redcedar increased from 0.08% to 18%, the most common tree species recorded, and post oak increased from 8% to 16%.

**Table 2:** Descending composition percentage (≥10%) of GLO line trees (encountered by the surveyor) and FIA trees (alive and ≥7.6 cm DBH) by subsections in the Missouri Ozarks.

	GLO line trees			FIA trees		
Subsection	Species	Count	%	Species	Count	%
Springfield Plain/OZ1	post oak	1933	30.65	post oak	528	19.32
Springfield Plain/OZ1	black oak	1601	25.38	black oak	499	18.26
Springfield Plain/OZ1	blackjack oak	1275	20.22	hickories	418	15.29
Springfield Plain/OZ1	hickories	609	9.66	blackjack oak	50	1.83
Central Plateau/OZ5	post oak	4205	44.15	post oak	1676	23.42
Central Plateau/OZ5	black oak	1783	18.72	black oak	1509	21.09
Central Plateau/OZ5	white oak	1576	16.55	white oak	1101	15.39
Central Plateau/OZ5	blackjack oak	1194	12.54	hickories	754	10.54
Central Plateau/OZ5	hickories	322	3.38	blackjack oak	319	4.46
White River Hills/OZ4	post oak	1444	32.83	eastern redcedar	1406	18.92
White River Hills/OZ4	black oak	1264	28.73	black oak	1384	18.62
White River Hills/OZ4	white oak	898	20.41	white oak	1040	13.99
White River Hills/OZ4	eastern redcedar	22	0.50	post oak	840	11.30
Osage River Hills/OZ6	black oak	1344	25.96	black oak	819	20.30
Osage River Hills/OZ6	white oak	1331	25.70	post oak	808	20.02
Osage River Hills/OZ6	post oak	1289	24.89	white oak	648	16.06
Osage River Hills/OZ6	eastern redcedar	3	0.06	eastern redcedar	388	9.62
Gasconade River Hills/OZ7	post oak	1316	31.30	black oak	814	20.32
Gasconade River Hills/OZ7	white oak	1182	28.12	white oak	784	19.57
Gasconade River Hills/OZ7	black oak	903	21.48	post oak	522	13.03
Gasconade River Hills/OZ7	hickories	175	4.16	shortleaf pine	420	10.48
Gasconade River Hills/OZ7	shortleaf pine	129	3.08	hickories	385	9.61
Meramec River Hills/OZ8	white oak	1680	49.28	white oak	1019	27.09
Meramec River Hills/OZ8	black oak	706	20.71	black oak	769	20.44
Meramec River Hills/OZ8	post oak	428	12.56	post oak	490	13.02
Current River Hills/OZ9	shortleaf pine	3849	54.43	white oak	2392	28.02
Current River Hills/OZ9	black oak	1130	15.98	black oak	2189	25.64
Current River Hills/OZ9	white oak	1088	15.38	shortleaf pine	1292	15.13
Current River Hills/OZ9	hickories	164	2.32	hickories	1030	12.06
Knobs and Basins/OZ10	white oak	1162	38.67	white oak	621	21.62
Knobs and Basins/OZ10	black oak	733	24.39	black oak	565	19.67
Knobs and Basins/OZ10	shortleaf pine	369	12.28	hickories	416	14.48
Knobs and Basins/OZ10	hickories	296	9.85	shortleaf pine	190	6.62
Black River Border/OZ14	white oak	1104	36.58	white oak	787	24.31
Black River Border/OZ14	black oak	755	25.02	black oak	598	18.47
Black River Border/OZ14	shortleaf pine	596	19.75	shortleaf pine	553	17.08
Black River Border/OZ14	hickories	160	5.30	post oak	456	14.08
Black River Border/OZ14	post oak	138	4.57	hickories	376	11.61
Outer Border/OZ12	white oak	3371	42.47	white oak	515	13.29
Outer Border/OZ12	black oak	2193	27.63	hickories	493	12.72
Outer Border/OZ12	hickories	895	11.27	eastern redcedar	424	10.94
Outer Border/OZ12	maples	151	1.90	black oak	402	10.37
Outer Border/OZ12	eastern redcedar	5	0.06	maples	390	10.06
Inner Border/OZ13	white oak	1669	43.76	eastern redcedar	525	17.50
Inner Border/OZ13	black oak	1079	28.29	post oak	475	15.83
Inner Border/OZ13	hickories	318	8.34	black oak	364	12.13
Inner Border/OZ13	post oak	294	7.71	hickories	346	11.53
Inner Border/OZ13	eastern redcedar	3	0.08	white oak	320	10.67

### Species Distribution Modeling

Predicted probabilities (possible values from 0 to 1) from species distribution models for GLO and FIA tree species groups were dissimilar and predicted probabilities varied by location (i.e., polygon). Correlation between the probabilities was low (mean  = 0.49), with a range from 0.81 for maples and blackgum to −0.03 for sassafras. True prediction rates were generally similar; at a threshold of 0.75, true positive rates ranged from 0.69 to 0.99 (mean  = 0.83) for GLO tree species groups and 0.42 to 0.96 (mean  = 0.81) for FIA tree species groups. Bur oak, sassafras, black oaks, white oak, hickories, and sycamore (*Platanus occidentalis*) had the greatest (>0.10) differences between GLO and FIA true positive rates.

### Species Distributions

**Figure 2:**
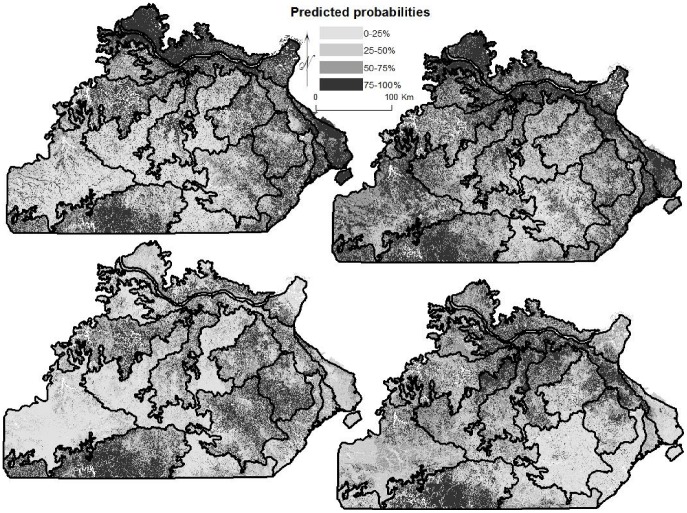
Predicted probabilities for species distributions of two fire-sensitive species. Panels are (a) historical distribution of elms, (b) current distribution of elms, (c) historical distribution of eastern redcedar, and (d) current distribution of eastern redcedar.

**Figure 3:**
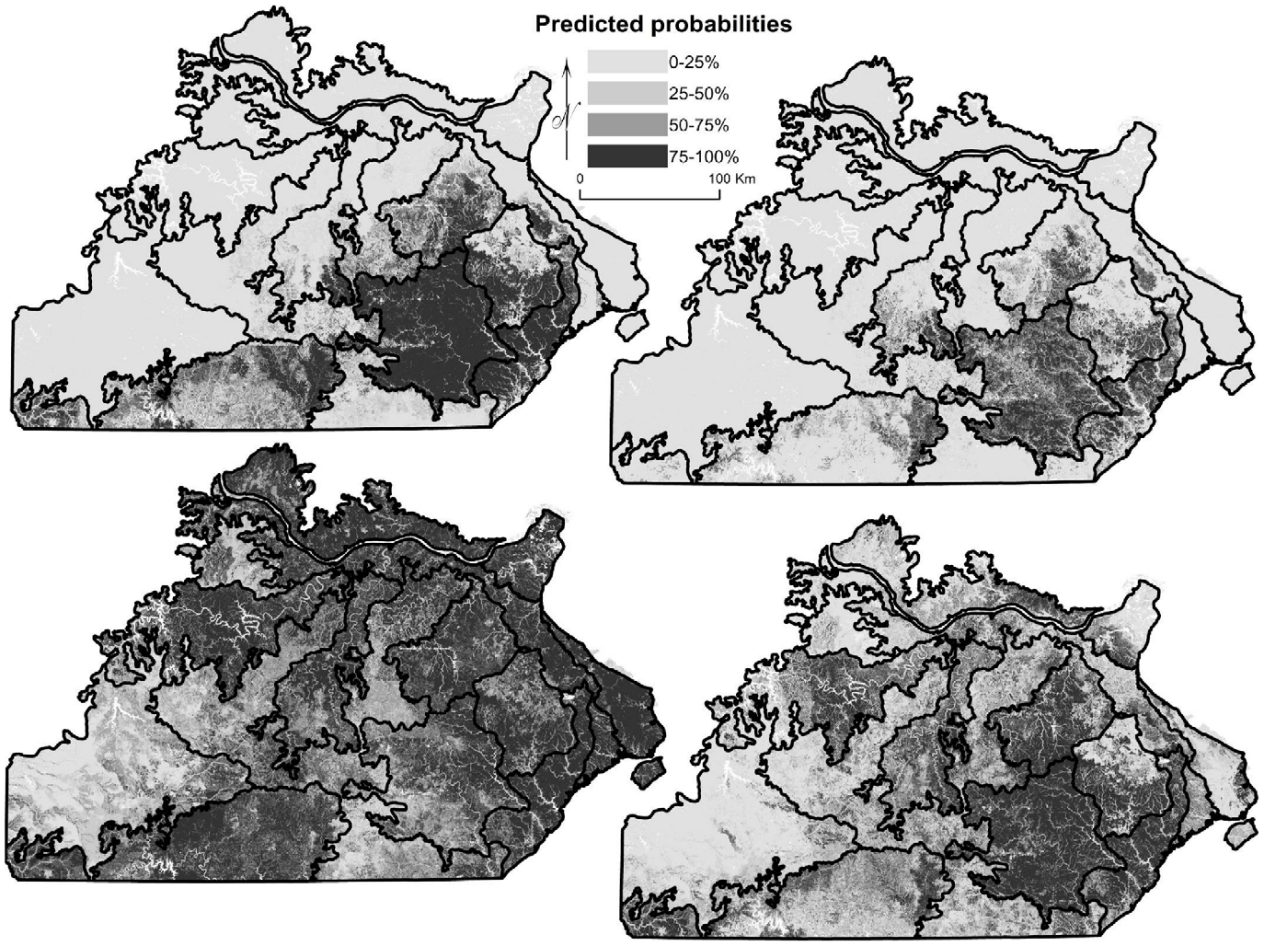
Predicted probabilities for species distributions of two fire-dependent species. Panels are (a) historical distribution of shortleaf pine, (b) current distribution of shortleaf pine, (c) historical distribution of white oak, and (d) current distribution of white oak.

Maps of species distributions displayed changes between historical and current forests, as most species exhibited reduced predicted probability in subsections where they were historically concentrated and increased predicted probability in more or all of the Missouri Ozarks, with shifts in distribution. Elms (Figure 2), ashes, walnuts, cherries, mesic species, maples, bottomland species, sycamore, sassafras, and hackberry (*Celtis occidentalis*) in general expanded from their most probable locations along the Inner and Outer Border subsections along the major rivers (Missouri and Mississippi; see Figure 1) to having increased probability throughout all or most subsections, and in some cases (e.g., cherries, sassafras, walnuts), a more probable presence in other subsections. Conversely, eastern redcedar (Figure 2) expanded from restricted glade areas and river bluffs within subsections to a strong presence along the Inner and Outer Border subsections. Hickories shifted from the exterior margins of the Ozarks to the interior. Chinkapin oak shifted from the western Ozarks to become equally probable in all subsections except the Central Plateaus. Bur oak shifted from the western Ozarks to become more probable along the rivers, albeit based on a small sample size (count of 42). The probability of presence decreased for shortleaf pine within its natural range in southern Missouri (Figure 3); contracted for post oak and blackjack oak shifting from central and western to central Ozarks; shifted for white oak from the northern and eastern half to being concentrated in southeastern Ozarks (Figure 3); and black oak experienced a contraction from all of the Ozarks to southeastern Ozarks. However, most species did increase in probability in one or more individual subsections.

### Species-environmental Relationships

The importance of environmental variables has changed over time, as twelve species or species groups shared two or fewer of the most influential five variables (of 24 total variables) for GLO and FIA models (Table 3). Bottomland species group, cherries, eastern redcedar, elms, hackberry, mesic species, sassafras, walnuts, white oak, bur oak, chinkapin oak, and pin oak shared one or two of the top five variables. The most common of the top five variables for GLO models were subsection (top five variable in 12 models; mean rank of 4.52 including all models), solar radiation (11 models), wetness index (11 models; mean rank was 5.67 including all models), elevation and parent material (9 models), and slope (8 models each). The most common of the top five variables for FIA models were subsection (top five variable in 14 models; mean rank of 3.14 including all models), pH and wetness index (10 models; mean rank of wetness index was 7.33 including all models), base saturation and taxonomic order (9 models each), and elevation (8 models).

**Table 3:** Top five predictor variables by rank (≥0.6) for GLO and FIA models and mean values using recorded tree location in the Missouri Ozarks.

Species group	Variable	GLO rank	FIA rank	GLO		FIA	
				×	SD	×	SD
ashes	elevation (m)	1.00	0.97	198.92	59.40	254.64	69.76
ashes	solar radiation	0.79	0.93	5669	75.35	5684	82.43
ashes	subsection	0.64	0.93				
ashes	taxonomic order		1.00				
ashes	geology		0.79				
black gum	subsection	1.00	1.00				
black gum	geology	0.82	0.54				
black oak	slope (%)	1.00	0.86	11.73	6.64	13.99	6.32
black oak	roughness	0.97	0.87	0.00	0.00	0.00	0.00
black oak	wetness	0.91	1.00	3.73	0.98	3.32	0.68
black oak	water content (cm/cm)	0.73		0.12	0.04	0.10	0.04
black oak	base saturation		0.80	0.63	0.29	0.53	0.18
black oak	pH		0.66	5.54	0.66	5.30	0.55
blackjack oak	elevation (m)	1.00	0.78	340.57	58.70	315.94	58.49
blackjack oak	subsection	0.93	1.00				
blackjack oak	solar radiation	0.85	0.99	5782	60.78	5759	58.37
bottomland	elevation (m)	1.00	0.75	160.67	37.39	177.60	51.74
bottomland	wetness		1.00	5.59	1.18	5.43	0.98
bur oak	wetness	1.00		5.52	1.21	4.15	1.06
bur oak	subsection	0.53	0.83				
bur oak	elevation (m)		1.00	260.70	58.19	242.80	43.16
bur oak	taxonomic order		0.78				
bur oak	base saturation		0.77	0.77	0.14	0.76	0.14
cherries	slope (%)	1.00	0.63	5.41	3.83	11.02	5.58
cherries	depth (cm)	0.69		167.19	39.88	124.22	58.82
cherries	parent material	0.65					
cherries	solar radiation		1.00	5713	55.29	5722	83.87
cherries	position		0.96	−0.16	0.65	0.04	1.01
cherries	roughness		0.88	0.00	0.00	0.00	0.00
cherries	subsection		0.73				
chinkapin oak	subsection	1.00	0.81				
chinkapin oak	slope (%)	0.38	0.65	16.60	8.12	15.69	6.58
chinkapin oak	pH		1.00	6.26	0.74	5.98	0.73
chinkapin oak	base saturation		0.80	0.78	0.16	0.73	0.18
eastern redcedar	slope (%)	1.00		19.56	7.48	13.19	5.76
eastern redcedar	roughness	0.67		0.0042	0.0024	0.0025	0.0018
eastern redcedar	subsection	0.56	0.80				
eastern redcedar	taxonomic order		1.00				
eastern redcedar	base saturation		0.95	0.80	0.18	0.73	0.18
eastern redcedar	pH		0.90	6.47	0.79	5.99	0.77
eastern redcedar	restriction		0.78				
elms	parent material	1.00					
elms	wetness	0.79	0.76	4.86	1.38	3.88	1.18
elms	origin	0.62					
elms	solar radiation	0.61		5686	63.50	5695	82.63
elms	subsection		1.00				
elms	pH		0.78	6.13	0.75	5.68	0.71
elms	taxonomic order		0.75				
elms	elevation (m)		0.74	223.03	62.20	267.36	74.11
hackberry	parent material	1.00					
hackberry	origin	0.69					
hackberry	wetness	0.41	0.71	5.43	1.19	4.51	1.44
hackberry	base saturation		1.00	0.85	0.21	0.75	0.17
hackberry	pH		0.98	6.39	0.78	6.02	0.67
hackberry	subsection		0.74				
hackberry	taxonomic order		0.67				
hickories	subsection	1.00	0.73				
hickories	geology	0.85					
hickories	slope (%)	0.67	0.86	9.33	6.17	13.16	6.19
hickories	wetness	0.63	0.83	4.17	1.16	3.49	0.84
hickories	base saturation		1.00	0.69	0.30	0.56	0.21
hickories	pH		0.80	5.74	0.68	5.38	0.63
maples	solar radiation	1.00	0.92	5657	73.40	5654	87.32
maples	subsection	0.97	1.00				
maples	elevation (m)	0.71	0.58	205.45	60.93	244.92	72.06
maples	landform	0.62					
maples	slope (%)	0.46	0.76	9.90	8.38	15.64	8.01
maples	roughness		0.71	0.0024	0.0027	0.0038	0.0027
mesic	elevation (m)	1.00	0.85	197.88	60.81	255.72	71.87
mesic	solar radiation	0.92		5674	61.16	5683	90.94
mesic	parent material	0.82					
mesic	subsection	0.52	1.00				
mesic	base saturation		0.88	0.79	0.21	0.68	0.18
mesic	position		0.82	−0.40	0.88	−0.33	1.09
mesic	wetness		0.78	4.93	1.38	3.93	1.13
pin oak	subsection	1.00					
pin oak	origin		1.00				
pin oak	organic (%)		0.81	1.09	0.75	0.57	0.15
pin oak	water content (cm/cm)		0.78	0.14	0.05	0.18	0.04
pin oak	depth (cm)		0.64	131.42	59.17	192.22	37.39
post oak	solar radiation	1.00	0.76	5757	70.54	5741	68.78
post oak	subsection	0.96	1.00				
post oak	elevation (m)	0.86		319.73	64.12	295.27	65.55
post oak	roughness	0.46	0.93	0.00	0.00	0.00	0.00
post oak	depth (cm)		0.76	102.14	59.55	99.83	56.98
post oak	wetness		0.71	3.78	0.81	3.49	0.61
sassafras	subsection	1.00	0.75				
sassafras	elevation (m)	0.95	0.60	194.87	63.77	294.76	78.46
sassafras	position		1.00	−0.13	0.73	0.13	1.21
sassafras	roughness		0.90	0.00	0.00	0.00	0.00
sassafras	slope (%)		0.65	10.82	9.17	13.08	6.44
shortleaf pine	subsection	1.00	1.00				
shortleaf pine	geology	0.70	0.57				
shortleaf pine	taxonomic order	0.66	0.63				
shortleaf pine	pH	0.60		5.11	0.36	5.11	0.39
sycamore	wetness	1.00	0.90	5.86	1.34	4.94	1.59
sycamore	landform	0.79	0.46				
sycamore	parent material	0.73					
sycamore	position		1.00	−0.69	1.19	−0.67	0.79
sycamore	pH		0.82	6.46	0.70	6.00	0.71
walnuts	parent material	1.00					
walnuts	wetness	0.81	0.91	4.95	1.49	3.99	1.16
walnuts	landform	0.78					
walnuts	base saturation	0.66	0.73	0.78	0.18	0.67	0.18
walnuts	solar radiation	0.63		5687	70.77	5710	85.36
walnuts	subsection		1.00				
walnuts	position		0.73	−0.49	0.99	−0.26	1.03
walnuts	pH		0.68	6.07	0.71	5.70	0.67
white oak	subsection	1.00					
white oak	elevation (m)	0.78		257.25	72.27	283.74	70.28
white oak	roughness	0.74	0.98	0.00	0.00	0.00	0.00
white oak	slope (%)	0.71	1.00	12.74	6.35	14.90	6.12
white oak	solar radiation	0.67		5692	79.27	5692	91.89
white oak	base saturation		0.83	0.63	0.33	0.51	0.18
white oak	pH		0.76	5.55	0.66	5.25	0.49
white oak	geology		0.69				

In general, species with increasing probabilities of presence were found on higher, drier, and sunnier sites, based on comparisons between the mean values of variables for trees recorded in GLO and FIA surveys (Table 3). Every species had at least a 12 meter change in mean elevation values. Blackjack, bur, chinkapin, pin, and post oaks showed increasing probabilities with lower elevations, generally 20 to 25 m less in elevation in modern times. Blackgum, eastern redcedar, hackberry, sassafras, shortleaf pine, and walnuts had greater probabilities in sunnier locations (increased mean values by at least 20 units), whereas blackjack oak and pin oak were most likely to occur in less sunny areas (decreased values by at least 20 units). Most species had decreased wetness index values, and ashes, blackgum, blackjack oak, bur oak, cherries, elms, hackberry, hickories, maples, mesic species group, sassafras, sycamore, and walnuts had values that changed at least 0.5 (for example, from 4.0 to 3.5) for the wetness index. Ashes, blackgum, black oak, blackjack oak, bur oak, cherries, elms, hackberry, hickories, maples, mesic species group, sassafras, sycamore, walnut, and white oak mean values for slope increased at least two percent units, whereas mean slope values for eastern redcedar and pin oak decreased at least two percent units. Most species had greater probabilities of presence on less basic soils (by at least 10% of GLO mean values), including ashes, blackgum, black oak, bottomland species group, cherries, eastern redcedar, elms, hackberry, hickories, maples, mesic species group, sassafras, sycamore, white oak, and walnuts.

### Communities along Environmental Gradients

**Figure 4:**
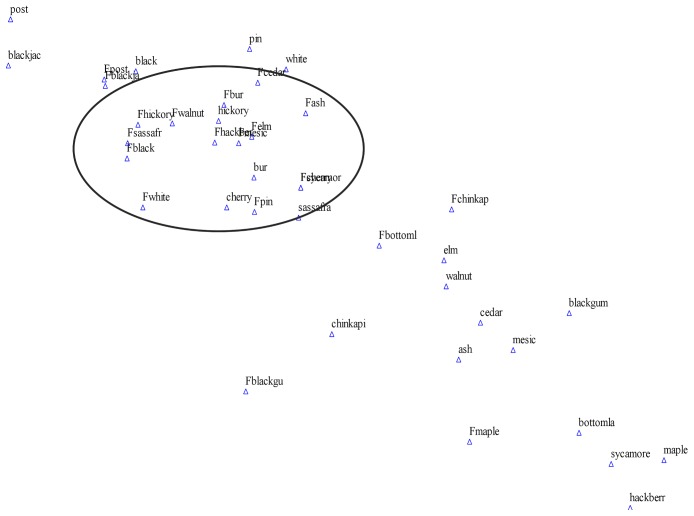
Ordination by historical and current species groups of Central Plateau ecological subsection in Missouri Ozarks. Current ecological units have prefix of ‘F’ and are centered within outlined circle, between historical post and blackjack oaks and the other historical species.

Although predicted probabilities of presence are not a measure of abundance, they do reflect response by species to environmental variables. Similar predicted probabilities for several species in an area may represent communities that have an affinity for a specific combination of environmental variables under a set disturbance regime. For historical forests, the NMS ordinations illustrated separation between the dominant and less common tree species; among bottomland, mesic, upland oak-hickory, and shortleaf pine associations; and individual species capable of inhabiting unique environments due their drought resistance (e.g., post oak and redcedar) or affinity with calcareous substrates (e.g., chinkapin oak and redcedar; Figure 4 for Central Plateau subsection example). Spatial arrangement of species and groups in the ordination plots varied among the subsections. Species groups in current forests displayed less departure among species and filled in the central spaces between the historical dominant and lesser species groups. The exception to this pattern was the Outer Border subsection, where the dominant tree species groups (white oak, black oak, and hickories) were relatively closer to a tightly concentrated group of mesic species (elms, walnuts, maples, and ashes) historically, compared to a relatively wider spread of species in current forests. The coefficient of determination for correlations between ordination distances and distances in the original n-dimensional space was no less than 0.966 for the Sorensen/Bray-Curtis distance measure. Stress, a measure of distance between original space and ordination space, and instability, standard deviation in stress over the final 10 iterations, were within normal limits; stress was no greater than 8.16 and instability was no greater than 0.00009.

## Discussion

### Regime Shift to an Alternative Stable State of Fire-sensitive Species Due to Weakened Effects of Gradients after Suppression of the Fire Regime

Succession to novel forests of fire-sensitive species in a region initially rich with a diverse mosaic of prairies, savannas, glades, woodlands, and forests has progressed in the Missouri Ozarks due to changes in fire regimes and land use [18], [20]. Historically, open oak ecosystems covered the landscape for thousands of years [36] despite climate variability (i.e., the Medieval Warm Period and Little Ice Age [37]); common oaks together with shortleaf pine and hickories accounted for most (93%) trees recorded by GLO surveyors. After fire cessation, the regime shift [38] to an alternative stable state of fire-intolerant species proceeded steadily [3], [39]–[41]. The black oak group, white oak, and post oak continued to be the most common species, however, they comprised only about half of the trees in modern inventories. Overall declines in oaks and shortleaf pine have been offset by increases in hickories and minor associates including fire-intolerant eastern redcedar, elms, and maples. At larger scales (sections and subsections), the landscape has become homogeneously mature oak-mixed species forests, even though at small scales forests are more diverse in tree species today than they were historically. However, surveyor bias for overstory, less shade-tolerant trees in GLO surveys may complicate comparisons with FIA surveys.

Ecological relationships among species distributions and environmental factors are not as distinct today as in historical times. Ordination of predicted probabilities from species distribution models displayed increased homogeneity among species distributions along environmental gradients. Ordinations of historical species showed greater distance along the x and y axes due to segregation of fire-dependent and fire-sensitive species along environmental gradients. Because fire is regulated by the same environmental variables, fire accentuated many of the relationships between tree species ecology and the environment. The specific fire regime determined which of a suite of potential species would dominate any given site over much of the landscape that is otherwise favorable for any number of species. Ordinations of species based on current surveys showed compressed clustering of species, indicating a decoupling of relationships between species and environmental variables and gradients. As opposed to early European settlers who maintained the use of fire, later human population expansion and development of the Ozark Highlands enforced fire suppression and changed long standing environmental-vegetation relationships in a formerly pyrogenic landscape. Similarly, Stambaugh and Guyette [19] observed weakened relationships between topographic roughness index and fire frequency following European settlement.

Physical factors such as temperature, wind, solar radiation and moisture vary along gradients both at local (valley to ridgetop and by aspect and slope position) and landscape (plains versus dissected hills) scales. Environmental gradients control fire disturbance that propagates across the land surface [19]. Interaction between environmental gradients and fire affects survival and reproduction of species, as well as fuel production and characteristics. Vegetation, as both the source of fuel and one of several determinants of microclimates, modifies the probability of fire ignition and behavior and ultimately, the fire regime. Likewise, lack of fire in areas that are fire-prone alter vegetation dynamics, species distributions, and dominance to diminish fundamental relationships between vegetation and environmental variables. In the Missouri Ozarks and eastern oak and pine forests, fire-sensitive species moved to previously fire-prone sites outside of protected, mesic areas that acted as firebreaks. Every species nevertheless maintained a unique distribution; thus different life history traits, such as amount of growth under different light and moisture levels, will influence survival along environmental gradients, even when not amplified by a fire regime.

### Environmental De-coupling of Distributions from Environmental Conditions

Patterns in species distribution at the scale of 10 to 1000 km^2^ (i.e., the subsection level) are influenced by differences in climate, geology, topography, soils, and hydrology, which are the underlying factors that define ecological subsections in Missouri [18]. Of the environmental variables for predicting species distributions, ecological subsection was a driving factor associated with species occurrence in the past and present. Ecological subsection became more influential for current species distributions, perhaps because stand-scale topographic variables that influenced fire regimes became less important without fire. Species continued to interact with their environment and expressed a pattern that became apparent at the subsection scale across the varied relief of the Ozark Highlands. However, because local factors have become less influential, eventually, the relationship between large spatial scales and tree species composition may deteriorate as fire-sensitive species continue dispersing and establishing throughout the now hospitable uplands. ‘Enduring’, or unique and representative, environmental features that form the current basis for conservation of associated biological diversity in some regions [42], may no longer define ecological communities with any permanence.

The majority of species were influenced by topographic variables, even though topography became less influential than in the past despite the rugged terrain of the Ozark Highlands. Fire-sensitive species were found on higher, drier, and sunnier sites, spatially consistent with increased presence in upland sites away from riparian associations. Changes to the list of species sensitive to topography occurred primarily with the minor species as they expanded from refugia in riparian environs (e.g., elm and walnut) or glades (e.g., eastern redcedar). The presence of eastern redcedar (high probability on steep slopes and areas of high roughness e.g., glade country in the White River Hills subsection) and walnut (high probability on bottomland landforms) originally was influenced by topographic variables, but these relationships weakened over time and now these species only have affinities for larger scale subsections, or spatial regions where conditions formerly were influential. Eastern redcedar is increasing its distribution rapidly (see species account below) and likely will establish beyond any spatial constraints in the future.

In historic times, the list of species that were sensitive to geologic and soil variables consisted of minor species and shortleaf pine. The potential range of shortleaf pine in Missouri is restricted by geology and soils because it only can compete with hardwoods on acidic, droughty, infertile soils derived from sandstone bedrock [43], especially in the absence of fire [44]–[45]. Walnuts are very sensitive to soil conditions requiring deep well-drained soils of good fertility (base saturation) and neutral pH [46]. Walnuts, elms, sycamore, and hackberry occurred primarily on alluvial parent materials in bottomlands. In the absence of fire and lessened importance of topographic relief, a more diverse list of species showed associations with geologic or soil variables. Black oak has increased substantially on former pine sites where soils are acidic and base saturation is low. Chinkapin oak and eastern redcedar are calciphiles; they can persist on high alkaline soils of high base saturation that form over carbonate bedrock, especially in areas with shallow soils such as dolomite glades. Soil taxonomic order and geology influenced ashes because they require high nitrogen and calcium fertility and soil moisture [46]. Soil depth influenced post oak, which is most competitive on shallow soils over bedrock or on flatwood sites where a fragipan limits soil depth.

### Shifts by Species Due to Loss of Disturbance

#### Oaks

White oak was dominant in the River Hills and most of the Ozark Border subsections, where the more dissected topography ameliorated the frequency of fire and the scale of fire disturbances [19]. White oak is 1) the most shade-tolerant of the oaks in this region, 2) more drought-tolerant than species in the black oak group, and 3) adapted to fire [47]–[48]. White oak is common in woodlands and forests where surface fires have reduced midstory canopies and created gap openings. White oaks have decreased by about 70% in the Ozark Border subsections along the Missouri and Mississippi Rivers because of strong competition by fire-intolerant species (eastern redcedar, maples) without the presence of fire and today, the core of white oak distribution primarily is in the River Hills of the eastern Ozarks. In the Current River Hills subsection, white oak almost doubled in abundance at the expense of shortleaf pine [49]. White oak continues to increase in dominance in this subsection as forests succeed to relatively shade-tolerant oak under a regime of fire suppression and partial cutting of the forest [50]. Regeneration of less shade-tolerant black oak and scarlet oak, and perhaps ultimately white oak, is not favored by retention of low to moderate overstory densities [51].

The black oak group was widely distributed and prominent throughout the Ozark Highlands, including the Springfield Plain and Central Plateau subsections, where black oak (*Q. velutina*) was present. Species in the black oak group vary in their physiology and ecology. Northern red and scarlet oak are more sensitive to fires, black and northern red oak are intermediate in their tolerance to shade, and scarlet oak is shade-intolerant. A 160% increase in the Current River Hills subsection was due to replacement of shortleaf pine by black oak and scarlet oak followed initial logging and burning of the pineries. Black oak and scarlet oak, like shortleaf pine, are strongly associated with acidic, well drained, droughty, intensely weathered (low fertility, low base cation) soils derived from Roubidoux sandstones or quaternary residuum on upper slope positions [20], [43]. Black oak and scarlet oak have suffered great mortality from oak decline recently in the Ozark Highlands due to the older age structure of forests that regenerated 70 to 100 years ago and occurrence of severe droughts on sites where *Armillaria* sp. are ubiquitous [51]–[52]. Nevertheless, drought has terminated oak stands for thousands of years and the establishment rather than mortality of oaks is problematic without fire. Following an oak decline event, a partial overstory remains under which advance reproduction of white oak and other more shade-tolerant species will recruit into the overstory without the presence of fire.

Post oak had a high probability of presence in the western and central Ozark Highlands due to drought tolerance and ability to persist as mature long-lived trees or oak grubs and seedling sprouts in a regime of frequent fires [23], [49]. In the Springfield Plain and Central Plateau subsections, post oak still is the most common species though it has been reduced by about 35 and 50% of its former presence, respectively. Post oak savannas were converted to pasture and today the flat subsections of central and southwestern Missouri are the heart of cattle and hay production. Fire suppression led to eastern redcedar encroachment in glades, savannas, and abandoned fields and increased forest density in remnant post oak woodlands, which prevented post oak establishment and favored succession to the competitors of shade-intolerant post oak.

Blackjack oak also was most common in the western and central Ozark Highlands. Blackjack oak is able to persist on xeric sites of low fertility, where it can find refugia from fire in rock outcrops, or where fire intensity and behavior is limited by discontinuous fuels and limited fine fuel production. For stems <15 cm in diameter, it is one of the more fire sensitive oaks [53]. Over the past 200 years, there has been a drastic loss, up to 90%, of blackjack oak throughout its historic range. Blackjack oak is slow-growing and shade-intolerant and consequently, it is easily overtopped and suppressed by faster growing vegetation and more shade-tolerant species. Conversion of post oak-blackjack oak savannas to pasture removed this species and invasion of glades by eastern red cedar and other hardwoods has increased competition intensity, making re-establishment difficult for blackjack oak without fire.

#### Shortleaf pine

Shortleaf pine is the only native pine in the Missouri Ozark Highlands and it is important both ecologically and economically. Periodic burning by Native Americans was spatially and temporally varied, which promoted pine regeneration and recruitment in the Missouri Ozarks (17,19–20). In addition to maintaining open forest understories and reducing competing vegetation, fire is needed to remove litter the layer for shortleaf pine germination on mineral soil or where litter is less than 3 cm deep [44]. Because shortleaf pines can live beyond 350 years, seed will fall on a favorable seed bed under a regime of periodic burning. However, frequent fire is a limiting factor because once pine seedlings are established, they need to become large enough either to resprout after burning or avoid shoot dieback altogether. Dey and Hartman [53] found that shortleaf pine advance reproduction that was at 4 cm in basal diameter had about a 60% chance of surviving a dormant season fire. Recruitment of shortleaf pine trees into the overstory requires a sufficiently long fire free period, which could vary from a minimum of 4 years for open-grown pines with average or better growth rates (1 cm diameter per year) to 20 years for pines growing under moderate levels of overstory shade or in small canopy gaps [45]. Therefore, Stambaugh et al. [45] concluded that a range in fire free interval from 8 to 15 years afforded adequate time for shortleaf pine seedlings to develop as large advance reproduction and to eventually recruit into the overstory. During European settlement of the Ozarks, fire frequency increased (e.g., every 3.5 years on average in the Current River Hills Region) compared to the Native American period (e.g, every 10 years on average; [17]). Additionally, settler fires ignited after logging to improve grazing conditions burned with greater intensity fueled by the slash from logging, thus increasing mortality in pine regeneration and residual mature trees. Oaks are better adapted to frequent fire than shortleaf pine [53] and shortleaf pine was relegated to the sites where it is most competitive, i.e., acid soils of low fertility and base saturation derived from sandstone bedrock [43].

Shortleaf pine continues to decline today in the absence of fire, with or without timber harvesting in Missouri and elsewhere throughout its range [54]. The range in Missouri was estimated at 2.7 million hectares before the logging era began in the late 1800 s [55] and currently, shortleaf pine is found on approximately 384,000 hectares of oak-pine forestlands [24]. The majority of forest lands in pine occur in the largest diameter classes and there is a deficiency in young shortleaf pine or oak-pine forests indicating a fundamental lack of sustainability [24]. Decades of fire suppression have allowed litter layers to accumulate inhibiting pine seedling establishment and promoting increases in forest density [24]. Low light levels in the understory cause high mortality in shortleaf pine seedlings that do establish; there is no chance to accumulate adequate numbers of pine advance reproduction. Any type of harvest without fire promotes succession to more shade-tolerant hardwoods such as white oak or sugar maple because there is no shortleaf pine advance reproduction and no establishment of pine after harvesting [44], [49].

#### Eastern redcedar

Eastern redcedar had little to no presence in the historical surveys across the Ozark Highlands and throughout the Midwest in the forest-prairie border region [56]–[57]. Eastern redcedar has several characteristics that make it sensitive to fire including thin bark and shallow roots and inability to resprout when the shoot is killed by fire. Trees less than 0.3 m tall are killed by any type of fire and more intense fires are needed to kill larger trees [58]–[59]. Batek et al. [20] found that eastern redcedar was common in the Missouri Ozarks where the historic, pre-European mean fire interval was ≥11 years, but it was absent where fires burned ≤5 years on average. Eastern redcedar occurred primarily in glades and along limestone river bluffs where it was protected from fire by rocky outcrops, bluffs, river breaks and discontinuous surface fuels [43], [60]. Eastern redcedar’s tolerance to drought, intolerance of shade, and ability to grow on high pH soils allow persistence on harsh, xeric, more open sites, especially calcareous soils.

We observed substantial expansion and dominance of eastern redcedar in the Missouri Ozark Highlands. In the White River Hills subsection, it became codominant with the black oak group and became a coleading species with post oak in the Inner Ozark Border subsection. In other subsections it rose from nearly non-existent to 10% of the surveyed trees. Blewett [56] quantified similar increases in eastern redcedar in the uplands along the Mississippi River in Iowa based on a comparison of GLO survey data and a current forest inventory. Schmidt and Leatherberry [61] reported that the acreage of eastern redcedar increased by about one million hectares in the lower Midwest from the 1960 s to 90 s and that 80% of the increase in eastern redcedar occurred in former pastures in Missouri. Eastern redcedar produces frequent large cone crops at an early age and seed is dispersed widely by birds [57], [62]. Seed from isolated, scattered trees and groves was sufficient to colonize glades, savannas, and abandoned pastures after fire was suppressed. Once eastern redcedar is established, the chance of fire spreading through the stand is greatly reduced because herbaceous plants are inhibited by shade and allelopathic mechanisms and fires are limited by lack of fine fuels and more mesic microenvironments [3], [57]. In addition, grazing of grasslands reduces the risk to burning, as does conversion of warm season grasslands to cool season grasses, both of which have promoted the invasion of eastern redcedar and other woody species by reducing fire frequency [41], [63].

#### Maples

Maples have been favored by fire suppression and forest management that produces shaded understories and small canopy gaps. In the Outer Ozark Border subsection, along the Missouri River, maple presence increased from 2 to 10%. Here, sugar maple replaced oaks on mesic sites with soils of high pH and base cation saturation [1]. Elsewhere in the Ozark Highlands, sugar maple regeneration and recruitment into the overstory has been reported on sites where soil pH is greater than 6.0, for example especially on Eminence and Gasconade Dolomite formations in the Current River Hills subsection [64]. In the most recent forest inventory in Missouri (2008), sugar maple has increased in all diameter size classes, especially in the >18 cm classes and currently more than 809,000 hectares have >2.3 m^2^/ha of sugar maple [24].

### Conclusions

Historical fire regimes integrated with the flat and dissected topographic relief of the Ozark Highlands to produce distribution patterns in tree species that were surveyed during the 1800 s. Where fire was relatively less frequent, for example in the heavily dissected areas of the Inner and Outer Border sections along the Missouri river, white oak dominated forests and where fires burned more frequently on western and central plains and plateaus, post and blackjack oaks prevailed in savannas and open woodlands such as on the Springfield Plain and Central Plateau subsections. Shortleaf pine and black oaks were dominant in areas of mild to moderate relief where fire regimes varied in frequency. Fire-sensitive species were relegated to wet or rocky refugia along river bluffs or glades.

Oaks were the dominant genus throughout the Ozark Highlands and although they still are today, there has been a major reduction in their dominance and distribution since the historical surveys, along with loss of open oak ecosystems. Species that expanded their distribution and presence in modern forests included eastern redcedar, hickories, elms, maples, chinkapin oak, ashes, and walnut. For now, most of these species or species groups are minor associates in mixed oak forests. Recruitment of fire-sensitive species occurred in areas with abiotic conditions that promoted fire, most notably eastern redcedar that in the past were restricted to rock outcroppings of glades and bluffs as well as a variety of fire-senstive species, which expanded from fire-protected subsections along riverine corridors to the uplands. Other researchers (e.g., [6], [65]–[66]) also have documented shifts from oak-dominated to mixed mesophytic communities in both upland and xeric sites. The net result of succession to forests and agricultural conversion and abandonment is that the landscape is more homogeneous in vegetation cover types, consisting of either eastern redcedar forests in former old fields or mature, closed canopied, fully-stocked hardwood forests [20], [56]. The suite of potential species that can dominate is more diverse with fire suppression and hence at finer spatial scales, upland forests have become more diverse.

Interactions among disturbance, environmental gradients, and functional traits of vegetation both determine and stabilize composition and distributions until a change occurs in the disturbance regime, creating new relationships between vegetation and gradients. Suppression of fire disturbance reduced stress on most species, which are not competitive in the presence of fire, allowing fire-sensitive species to spread throughout the varied relief away from firebreaks. Functional traits for fire tolerance formerly limited species to complementary environmental gradients, particularly topographic gradients in areas of greater relief such as the Ozark Highlands, but without fire, environmental gradients do not limit pioneering and mesic species establishment. Vegetation then reinforces site conditions; establishment by fire-sensitive species makes sites more fire-resistant in eastern forests, thus gradually favoring species that are shade-tolerant and will continue to stabilize mesic site conditions (i.e., the process of mesophication; [3]).

Interspecies interactions for light, water, and growing space is driving the composition of future forests without fire and land use disturbance that supports open oak and pine ecosystems. Interspecies interactions have become more important than abiotic factors because fire suppression has decoupled tree distributions from environmental gradients. Fundamental ecological principles that applied to past species distributions are no longer as applicable, which challenges the basis of models developed to describe present and future conditions. New model development, analysis of existing data, projections of climate change impacts, and conservation strategies are necessary to incorporate modern interactions among species in the absence of disturbance.

The processes of 1a) disturbance by fire and alternatively 1b) disturbance by grazing and harvest in open ecosystems or 2) competition in progressively dense forests drive and reinforce different forest conditions in the Ozark Highlands and elsewhere. Open oak and pine forests represent a unique situation, where anthropogenic disturbance was preventing classical succession to an alternative stable state. Shifting forest species composition due to successional replacement of oak species by more fire-sensitive and increasingly shade-tolerant competitors continues to occur without fire [3], [9]–[11], [47]–[48]. The rapid regime shift from open oak and pine savannas and woodlands to homogenous mixed oak forests during the past 100 years should proceed to denser forests of shade-tolerant species, given continued lack of disturbance. Novel communities are emerging due to changes in human land use and management. The trajectory of these forests is to an alternative stable state because disturbance-sensitive species reduce the probability of fire and drought and thus, reinforce lack of disturbance. Replacement by late-successional forests in upland oak ecosystems across the eastern United States and world-wide has consequences for landscape diversity and associated understory plants and wildlife.

## References

[1] Nigh TA, Pallardy SG, Garrett HE (1985). Sugar maple-environment relationships in the River Hills and central Ozark Mountains of Missouri.. Am Midl Nat.

[2] Pallardy SG, Nigh TA, Garrett HE (1988). Changes in forest composition in central Missouri: 1968–1982.. Amer Midl Nat.

[3] Nowacki GJ, Abrams MD (2008). The demise of fire and ‘mesophication’ of forests in the eastern United States.. Bioscience.

[4] Foster DR, Motzkin G, Slater B (1998). Land-use history as long-term broad scale disturbance: regional forest dynamics in central New England.. Ecosystems.

[5] Fuller JL, Foster DR, McLachlan JS, Drake N (1998). Impact of human activity on regional forest composition and dynamics in central New England.. Ecosystems.

[6] Surrette SB, Aquilani SM, Brewer JS (2008). Current and historical composition and size structure of upland forests across a soil gradient in north Mississippi.. Southeast Nat.

[7] Iverson LR, Dale ME, Scott CT, Prasad A (1997). A GIS-integrated moisture-index to predict forest composition and productivity in Ohio forests (U.S.A.).. Landscape Ecol.

[8] DeSantis RD, Hallgren SW, Lynch TB, Burton JA, Palmer MW (2010). Long-term directional changes in upland *Quercus* forests throughout Oklahoma, USA.. J Veg Sci.

[9] Lorimer CG (1984). Development of the red maple understory in northeastern oak forests.. For Sci.

[10] Abrams MD (1998). The red maple paradox.. BioSci.

[11] Fei S, Steiner KC (2007). Evidence for increasing red maple abundance in the eastern United States.. For Sci.

[12] Adams DE, Anderson RC (1980). Species response to a moisture gradient in central Illinois forests.. Amer J Bot.

[13] Abrams MD, Ruffner CM (1995). Physiographic analysis of witness-tree distribution (1765–1798) and present forest cover through north central Pennsylvania.. Can J For Res.

[14] Cowell CM, Hayes JJ (2007). Structure, history, and dynamics of a mature oak-beech forest in western Indiana.. J Torrey Bot Soc.

[15] Amatangelo KL, Fulton MR, Rogers DA, Waller DM (2011). Converging forest community composition along an edaphic gradient threatens landscape-level diversity.. Divers Distrib.

[16] Nelson JC (1997). Presettlement vegetation patterns along the 5^th^ Principal Meridian, Missouri Territory, 1815.. Am Midl Nat.

[17] Guyette RP, Muzika RM, Dey DC (2002). Dynamics of an anthropogenic fire regime.. Ecosystems.

[18] Nigh TA, Schroeder WA (2002). Atlas of Missouri ecoregions. Missouri: Missouri Department of Conservation Publication.. 212 p.

[19] Stambaugh MC, Guyette RP (2008). Predicting spatio-temporal variability in fire return intervals using a topographic roughness index.. For Ecol Manage.

[20] Batek MJ, Rebertus AJ, Schroeder WA, Haithcoat TL, Compas E (1999). Reconstruction of early nineteenth-century vegetation and fire regimes in the Missouri Ozarks.. J Biogeogr.

[21] Chapman CH, Chapman EF (1983). Indians and archaeology of Missouri. Revised edition.. Columbia, MO: University of Missouri Press.

[22] Guyette RP, Spetich MA, Stambaugh MC (2006). Historic fire regime dynamics and forcing factors in the Boston Mountains, Arkansas, USA.. For Ecol Manage.

[23] Guyette RP, Stambaugh MC (2004). Post oak fire scars as a function of diameter, growth, and tree age.. For Ecol Manage.

[24] Raeker G, Moser WK, Butler BJ, Fleming J, Gormanson DD (2011). Missouri’s forests 2008.. USDA For Serv Resourc Bull.

[25] White CA (1983). A History of the Rectangular Survey System.. Washington DC: Bureau of Land Management, Government Printing Office.774 p.

[26] Bourdo EA (1956). A review of the General Land Office survey and of its use in quantitative studies of former forests.. Ecology.

[27] Beers TW, Dress PE, Wensel LC (1966). Aspect transformation in site productivity research.. J For.

[28] Sappington JM, Longshore KM, Thompson DB (2007). Quantifying landscape ruggedness for animal habitat analysis: a case study using bighorn sheep in the Mojave Desert.. J Wildl Manage.

[29] Breiman L (2001). Random Forests.. Mach Learn.

[30] Cutler DR, Edwards, TC, Beard KH, Cutler A, Hess KT (2007). Random forests for classification in ecology.. Ecology.

[31] Ridgeway G (1999). The state of boosting.. Comput Science and Stat.

[32] Friedman J, Hastie T, Tibshirani R (2000). Additive logistic regression: a statistical view of boosting.. Ann Stat.

[33] Elith J, Leathwick JR, Hastie T (2008). A working guide to boosted regression trees.. J Anim Ecol.

[34] Liaw A, Wiener M (2002). Classification and Regression by randomForest.. R News.

[35] Sing T, Sander O, Beerenwinkel N, Lengauer T (2005). ROCR: visualizing classifier performance in R. Bioinformatics.

[36] Stambaugh M, Guyette RP (2009). Progress in constructing a long oak chronology from the central United States.. Tree-ring Res.

[37] Umbanhowar Jr CE, Camill P, Geiss CE, Teed R (2006). Asymmetric vegetation responses to mid-Holocene aridity at the prairie–forest ecotone in south-central Minnesota.. Quaternary Res.

[38] Scheffer M, Carpenter SR (2003). Catastrophic regime shifts in ecosystems: linking theory to observation.. Trends Ecol Evol.

[39] Cottam G (1949). The phytosociology of an oak woods in southwestern Wisconsin.. Ecology.

[40] Grimm EC (1984). Fire and other factors controlling the Big Woods vegetation of Minnesota in the mid-nineteenth century.. Ecol Monogr.

[41] Briggs JM, Knapp AK, Brock BL (2002). Expansion of woody plants in tallgrass prairie: a fifteen-year study of fire and fire-grazing interactions.. Am Midl Nat.

[42] Lemieux CJ, Scott DJ (2005). Climate change, biodiversity conservation and protected area planning in Canada.. Canadian Geogr.

[43] Fletcher PW, McDermott RE (1957). Influence of geologic parent material and climate on distribution of shortleaf pine in Missouri.. Columbia, MO: Univ Missouri College Agric Agric Exp Sta Res Bull 625.

[44] Stambaugh MC, Muzika R-M (2007). Successional trends of six mature shortleaf pine forests in Missouri.. USDA For Serv Northern Res Sta Gen Tech Rep.

[45] Stambaugh MC, Guyette RP, Dey DC (2007). What fire frequency is appropriate for shortleaf pine regeneration and survival?. USDA For Serv Northern Res Sta Gen Tech Rep.

[46] Burns RM, Honkala BH (1990). Silvics of North America Volume 2: Hardwoods.. Washington, DC: USDA For Serv Agric Handb.

[47] Abrams MD (1992). Fire and the development of oak forests in eastern North America.. BioSci.

[48] Abrams MD (2003). Where has all the white oak gone?. BioSci.

[49] Pallardy SG (1995). Vegetation analysis, environmental relationships, and potential successional trends in the Missouri forest ecosystem project.. USDA For Serv Gen Tech Rep.

[50] Kabrick JM, Zenner EK, Dey DC, Gwaze D, Jensen RG (2008). Using ecological land types to examine landscape-scale oak regeneration dynamics.. For Ecol Manage.

[51] Kabrick JM, Dey DC, Jensen RG, Wallendorf M (2008b). The role of environmental factors in oak decline and mortality in the Ozark Highlands.. For Ecol Manage.

[52] Bruhn JN, Wetterhoff JJ, Mihail JD, Kabrick JM, Pickens JB (2000). Distribution of *Armillaria* species in upland Ozark Mountain forests with respect to site, overstory species composition and oak decline.. Eur J For Path.

[53] Dey DC, Hartman G (2005). Returning fire to Ozark Highland forest ecosystems: Effects on advance regeneration.. For Ecol Manage.

[54] South DB, Buckner ER (2003). The decline of southern yellow pine timberland.. J For.

[55] Liming FG (1946). The range and distribution of shortleaf pine in Missouri.. USDA Cent States For Exp Sta Tech Paper No 106.

[56] Blewett TJ (1986). Eastern redcedar’s (*Juniperus virginiana* L.) expanded role in the prairie-forest border region.. Proc 9^th^ North American Prairie Conf.

[57] Norris MD, Blair JM, Johnson LC (2001). Land cover change in eastern Kansas: litter dynamics of closed-canopy eastern redcedar forests in tallgrass prairie.. Can J Bot.

[58] Stritzke JF, Bidwell TG (1990). Eastern redcedar and its control.. Oklahoma State Univ Ext Facts F-2850.

[59] Engle DM, Stritzke JF (1995). Fire behavior and fire effects on eastern redcedar in hardwood leaf-litter fires.. Int J Wildland Fire.

[60] Kucera CL, Martin SC (1957). Vegetation and soil relationships in the glade region of the southwestern Missouri Ozarks.. Ecol.

[61] Schmidt TL, Leatherberry EC (1995). Expansion of eastern redcedar in the lower Midwest.. North J Appl For.

[62] Holthuijzen AMA, Sharik TL, Fraser JD (1987). Dispersal of eastern red cedar (*Juniperus virginiana*) into pastures: an overview.. Can J Bot.

[63] Schmidt TL, Stubbendieck J (1993). Factors influencing eastern redcedar seedling survival on rangeland.. J Range Manage.

[64] Ware S, Redfearn Jr PL, Pyrah GL, Weber WR (1992). Soil pH, topography and forest vegetation in the central Ozarks.. Amer Midl Natur.

[65] Rentch JS, Fajvan MA, Hicks RR (2003). Oak establishment and canopy accession strategies in five old-growth stands in the central hardwood forest region.. For Ecol Manage.

[66] Ozier TB, Groninger JW, Ruffner CM (2006). Community composition and structural changes in a managed Illinois Ozark Hills forest.. Amer Midl Nat.

